# Beef intake and risk of rheumatoid arthritis: Insights from a cross-sectional study and two-sample Mendelian randomization

**DOI:** 10.3389/fnut.2022.923472

**Published:** 2022-09-06

**Authors:** Weiwei Chen, Ke Liu, Lin Huang, Yingying Mao, Chengping Wen, Ding Ye, Zhixing He

**Affiliations:** ^1^Department of Epidemiology, School of Public Health, Zhejiang Chinese Medical University, Hangzhou, Zhejiang, China; ^2^Institute of Basic Research in Clinical Medicine, School of Basic Medical Sciences, Zhejiang Chinese Medical University, Hangzhou, Zhejiang, China

**Keywords:** beef, rheumatoid arthritis, NHANES, cross-sectional, Mendelian randomization

## Abstract

**Background:**

Beef is common in daily diet, but its association with the risk of rheumatoid arthritis (RA) remains uncertain. The objective of this study is to explore the relationship between beef intake and the risk of RA.

**Materials and methods:**

We investigated the association between beef intake and risk of RA by multivariate logistic regression, based on the National Health and Nutrition Examination Survey (NHANES) 1999–2016 involving 9,618 participants. The dose–response relationship between beef intake and RA was explored as well. Furthermore, we performed Mendelian randomization (MR) analysis to examine the causal effect of beef intake on RA. Genetic instruments for beef intake were selected from a genome-wide association study (GWAS) including 335,576 individuals from the UK Biobank study, and summary statistics relating to RA were obtained from a GWAS meta-analysis of 14,361 RA patients and 43,923 controls. The inverse-variance weighted (IVW) approach was used to estimate the causal association, and MR-Egger regression and Mendelian randomization pleiotropy residual sum and outlier (MR-PRESSO) test were applied to evaluate the pleiotropy and outliers.

**Results:**

Compared with the lowest quintile (0 to ≤33.50 g/d), beef intake was found to be significantly associated with the risk of RA [odds ratio (OR): 1.94; 95% confidence interval (CI): 1.20–3.12] in the third quintile (50.26 to ≤76.50 g/d). Moreover, a reversed “U” dose–response relationship between beef and RA (*P*_non–linearity_ = 0.023) was found. In the MR analysis, beef intake was associated with an increased risk of RA (OR: 3.05; 95% CI: 1.11–8.35; *P* = 0.030) by the IVW method. The results from MR-Egger regression and MR-PRESSO test showed that there were no pleiotropic variations and outliers.

**Conclusion:**

This study indicated that there is suggestive evidence to support the causal effect of beef intake on the risk of RA, while further studies are warranted to elucidate the exact association.

## Introduction

Rheumatoid arthritis (RA) is a chronic systemic autoimmune disease that corrodes arthrosis and causes progressive articular damage (1). The annual incidence rate of RA was 14.9% in 2017, which had increased by 8.2% compared with that in 1990 around the world (2). It was estimated that 20–30% of RA patients would be invalidity for work permanently without any medical treatment within 2–3 years of diagnosis (3).

Accumulating risk factors have been found to play an important role in RA, such as smoking, breastfeeding, silica exposure, and educational level (4–7). Recently, it has received growing attention that dietary patterns and nutrients are potentially modifiable factors affecting the occurrence and development of RA (8–10). A Mediterranean diet is recommended to prevent the occurrence and complications of RA, due to the abundance of antioxidants and anti-inflammatory foods (11). In contrast, a western diet that contains high consumption of red meat and saturated fat may be associated with a high risk of RA by directly causing inflammation or indirectly raising insulin resistance and body mass index (BMI) (12–14). However, the role of red meat in the risk of RA remains controversy. For example, a large cohort study consisting of 80,551 post-menopausal women in the United States suggested that high red meat intake was associated with an elevated risk of RA (15). However, a 22-year Nurses’ Health Study (NHS) cohort study (16), which collected diet information from 82,063 participants by semi-quantitative food frequency questionnaires (FFQ), showed that there was no significant association between red meat intake and RA risk. One reason that could explain the discrepancy in findings may be due to the differences in the composition of red meat in previous studies, since different types of red meat have different nutritional contents, which may lead to different health outcomes and risk of diseases (17, 18). Beef, is a major source of red meat, although it provides various nutrients that are essential to humans (19, 20), its high protein and fat content hold the potential to increase the risk of RA (21). Nevertheless, limited epidemiological studies have explored the association between beef consumption and the risk of RA.

To be noted, diets are associated with a variety of clinical and social factors, and it is difficult to assess the causal effects of diets on multiple outcomes. Mendelian randomization (MR) is an analytical method that assesses the causal association between exposure and outcome by introducing genetic instrumental variables (IVs), such as single nucleotide polymorphisms (SNPs) (22). Since IVs are independent of other traits and are inherited randomly, MR analysis can largely reduce the interference of confounding factors and reduce the possibility of reverse causality (23, 24). This approach is increasingly applied in assessing and screening potentially causal associations (25–27), which would be useful to detect the causal effect of beef intake on the risk of RA.

In this study, we first conducted a cross-sectional study based on the National Health and Nutrition Examination Survey (NHANES) to determine the observational association between beef intake and the risk of RA. Then, we further implemented MR analysis to assess the causal relationship between beef intake and the risk of RA.

## Materials and methods

### Cross-sectional study

#### Study population in National Health and Nutrition Examination Survey

The NHANES is a cross-sectional survey designed to assess the health and nutritional status of Americans, and it has been a continuous program since 1990 and is updated every 2 years (28). In this current study, we combined data from 1999 to 2016 to increase the sample size. We included non-Hispanic whites aged more than 20 years and excluded the participants with missing information of covariates. All study participants supplied the written informed consent and the study was approved by National Center for Health Statistics Research Ethics Review Board.

#### Beef consumption and rheumatoid arthritis assessment

Participants were asked to complete two 24-h dietary recalls for each cycle except only once in the 1999–2000 wave. Each food consumption was assigned an 8-digit Food and Nutrient Database for Dietary Studies (FNDDS) code and the code for beef products was 21000000–21800000 (29). We assessed the beef intake by calculating the sum of the weight of all beef products consumed by participants over a 24-h dietary recall. For RA assessment, the participants were asked two questions about RA: (1) Have doctors ever said they had arthritis? (2) Which type of arthritis? If participants answered yes to the first question and answered “rheumatoid arthritis” to the second question, then he/she would be considered RA patients. Otherwise, he/she was considered a non-RA individual.

#### Statistical analysis

Weighted analysis was conducted using the sample weights, stratification, and clustering variables to account for the complex sampling design in NHANES. In this study, we rebuilt a new 18-year dietary weight because of combining nine 2-year survey cycles of NHANES.^1^

The multivariate logistic regression was applied to estimate the odds ratio (OR) and 95% confidence interval (CI) for the association between beef intake and risk of RA. First, we explored the effect of beef intake (none and yes) on RA independently. Second, we investigated the association of beef consumption with RA by categorizing beef consumption into quintiles (Q1, 0 to ≤33.50 g/d; Q2, 33.50 to ≤50.26 g/d; Q3, 50.26 to ≤76.50 g/d; Q4, 76.50 to ≤118.00 g/d; Q5, >118.00 g/d). Two sets of adjusting covariates were constructed in the logistic regression model. Model 1 was assembled by adjusting for age, sex, education, poverty-income rate, and marriage. In addition to the factors adjusted in model 1, smoking, alcohol drinking, history of diabetes, and BMI (kg/m^2^) were considered in model 2. Furthermore, the method described by Greenland and Longnecker (30) was used to estimate the dose–response relationship. For the highest dose group, the lower limit plus the width of the previous group was supposed as the corresponding beef consumption. The other dose groups were assigned the midpoint of the lower and upper bound.

Statistical analyses were performed by SAS version 9.4 and *P* < 0.05 was regarded as statistically significant.

### Mendelian randomization study

#### Summary dataset of rheumatoid arthritis

The genetic association data of RA was obtained from a genome-wide association study (GWAS) meta-analysis of 14,361 RA cases and 43,923 controls of European ancestry (31). A total of 42 loci were identified to be significantly associated with RA at the genomic level (*P* < 5×10^−8^). More information and details about this study have been reported in the previous article (31). The written informed consent was provided by all study participants and the study was allowed by each local agency review board.

#### Selection of beef intake associated single nucleotide polymorphisms

Beef intake-associated SNPs were selected from a large-scale GWAS based on 335,576 individuals of white European descent from the UK Biobank study (32). Beef consumption was assessed according to a diet questionnaire by asking “How often do you eat beef?” and a competitive analysis was used to test the association between genotype and phenotype (32). Supplementary Table 1 lists the details of the GWAS studies and datasets used in the MR study. A total of seven loci were associated with beef intake at the genome-wide significant threshold (*P* < 5×10^−8^). All of them were not in linkage disequilibrium (*r*^2^ < 0.1) and not overlapped with the known risk of RA (33). However, one SNP (rs66495454) was eliminated because it was not found in the outcome GWAS, thus six SNPs were used as IVs. The details of instrumental SNPs in this study are shown in Supplementary Table 2.

Furthermore, to assess the strength of the IVs, the *F*-statistics were calculated by the formula of *F* = *R*^2^×(*N*−*k*−1)/*k*×(1−*R*^2^) (34), where *R*^2^ is the total variance explained by the IVs, *N* represents the sample size and *k* indicates the number of included IVs. The variance of each IV was computed by minor allele frequency (MAF) and β value (35). In addition, the statistical power of MR analysis to detect causal association was calculated (36).

#### Statistical analysis

The inverse-variance weighted (IVW) method was used to evaluate the causal association between beef intake and the risk of RA. The IVW method performs a meta-analysis of Wald values (i.e., the beta coefficient of the SNP for outcome divided by the beta coefficient of the SNP for exposure) to estimate the overall causal association between exposure and outcome (37). In addition, the maximum-likelihood method was used to validate the result from the IVW method, which is assessed by assuming that there was a linear relationship between beef intake and risk of RA (38).

Then we used MR-Egger regression to assess potential directional pleiotropy by checking the intercept term. It indicates that directional pleiotropy may not exist when the intercept term was close to zero (39). Moreover, to evaluate the horizontal pleiotropy level of the IVs, Mendelian randomization pleiotropy residual sum and outlier (MR-PRESSO) were employed, which is comprised of three parts [(a) detection of horizontal pleiotropy, (b) correction by removal of offending IVs, and (c) test of significant differences in the causal estimates before and after removal of outlier] (40). Furthermore, we used Cochran’s *Q* test to estimate the consistency of the association between beef intake and the risk of RA across each IV.

Furthermore, the GWAS Catalog^2^ was searched to find whether the instrumental SNPs were related to other traits. We also conducted sensitivity analysis by the “leave-one-out” method to assess the reliability of causality. We eliminated each SNP one by one and combined the effect value of the remaining. The fluctuation of the results before and after removing the SNP reflects the stability of the association.

Statistical analyses were performed by using R version 4.0.5 and *P* < 0.05 was regarded as statistically significant.

## Results

### Cross-sectional study

**FIGURE 1:**
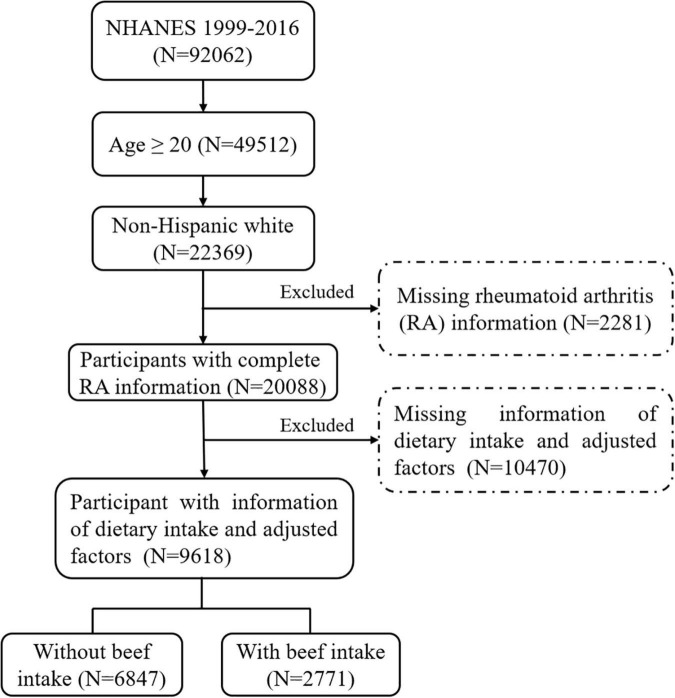
The flow diagram of participant selection.

The details of the inclusion and exclusion criteria of subjects are shown in Figure 1. Consequently, a total of 9,618 participants were eventually included in this cross-sectional study. Compared with non-RA individuals, patients with RA seemed to have higher BMI and lower poverty-income ratio. The detailed characteristics of the included participants are presented in Table 1.

**TABLE 1:** Baseline characteristics of selected participants from National Health and Nutrition Examination Survey (NHANES) 1999–2016.

Characteristics	RA (*N* = 906)	Non-RA (*N* = 8712)
**Age**		
20∼65 years	430 (47.46%)	6510 (74.72%)
≥65 years	476 (52.54%)	2202 (25.28%)
**Sex**		
Female	480 (52.98%)	4027 (46.22%)
Male	426 (47.02%)	4685 (53.78%)
BMI (kg/m^2^)	29.73 ± 7.31	27.84 ± 6.21
Poverty-income ratio	2.75 ± 1.66	3.09 ± 1.65
**Education**		
Less than high school	243 (26.82%)	1717 (19.71%)
High school graduate	348 (38.41%)	3122 (35.84%)
More than high school	315 (34.77%)	3873 (44.46%)
**Married**		
Yes	527 (58.17%)	5189 (59.56%)
No	379 (41.83%)	3523 (40.44%)
**Diabetes**		
Yes	154 (17.00%)	636 (7.30%)
No	752 (83.00%)	8076 (92.70%)
**Smoked at least 100 cigarettes in life**		
Yes	585 (64.57%)	4816 (55.28%)
No	321 (35.43%)	3896 (44.72%)
Frequency of alcohol drinks in the past 12 months, median (IQR)	1 (0, 3)	2 (1, 3)

IQR, interquartile range; RA, rheumatoid arthritis.

**FIGURE 2:**
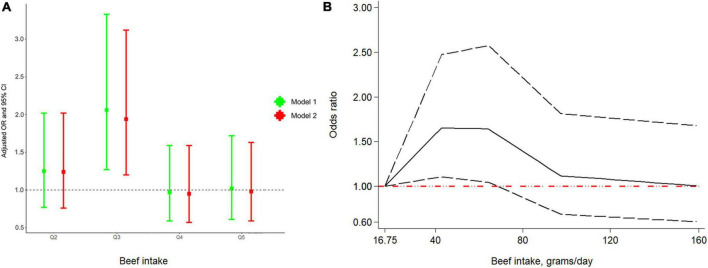
Odds ratio (OR) between quintiles of beef intake and RA **(A)** and dose-response relationship between beef intake per day and RA **(B)**. Abbreviation: RA, rheumatoid arthritis.

Even though ever beef intake was not significantly associated with the risk of RA (Supplementary Table 3), we found that the risk of RA in the third quintile was 2.06 times than in the first quintile (OR: 2.06; 95% CI 1.27–3.33) in model 1 (Figure 2A). Similarly, in model 2, the association between beef and RA remained robust (OR: 1.94; 95% CI: 1.20–3.12). Additionally, as depicted in Figure 2B, there was an interesting non-linear relationship between RA and beef (*P*_non–linearity_ = 0.023). In particular, an increased risk of RA was observed when beef intake ranged from 16.75 to 68.67 g/day.

### Mendelian randomization study

**FIGURE 3:**
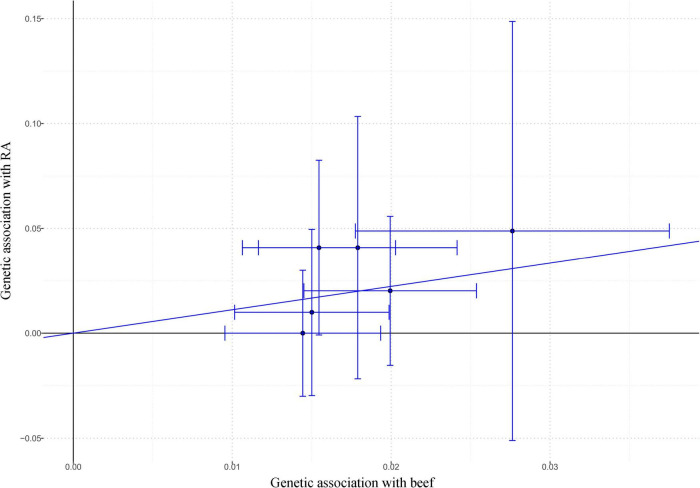
The effect size and 95% CI of each SNP on the association between beef intake and RA risk by IVW. Abbreviations: CI, confidence interval; IVW, inverse-variance weighted; RA, rheumatoid arthritis; SNP, single nucleotide polymorphism.

As shown in Figure 3, beef intake was associated with an increased risk of RA (OR: 3.05; 95% CI: 1.11–8.35; *P* = 0.030) by the IVW method. Similarly, genetically predicted beef intake was positively associated with the risk of RA by the maximum-likelihood method (OR: 3.12; 95% CI: 1.10–8.79; *P* = 0.032). There was no indication for directional pleiotropy effects (*P* = 0.547) as assessed by the MR-Egger intercept (Table 2). Also, there was no evidence for heterogeneity (*P* = 0.698) in the association of any IV with the risk of RA as measured by the Cochran’s *Q* test, and no outlier SNPs (*P* = 0.708) were detected with the MR-PRESSO test (Table 2). The *F*-statistics ranged from 30.05 to 51.82, suggesting the IVs were unlikely to be affected by weak instruments (Supplementary Table 2). Statistical power was calculated to be 89.77% to detect an effect size of 3.05 at a significance level of 0.05.

**TABLE 2:** A causal association between intake of beef and risk of rheumatoid arthritis (RA).

Outcomes and methods	Number of SNPs	OR	95% CI	*P* for association	*P* for Cochran’s *Q* test	*P* for MR-PRESSO global test
IVW (fixed)	6	3.05	1.11–8.35	0.030	0.698	
MR-Egger	6	/	/	0.547^a^		
Maximum-likelihood	6	3.12	1.10–8.79	0.032		
MR-PRESSO (0 outliers)	6	3.05	1.40–6.66	0.038		0.708

^a^*P*-value for the intercept of MR-Egger regression analysis.

CI, confidence interval; IVW, inverse-variance weighted; MR-PRESSO, Mendelian randomization pleiotropy residual sum and outlier; OR, odds ratio; SNP, single nucleotide polymorphism.

Of the six IVs used in MR analysis, there were four SNPs statistically correlated with different traits, such as rs4676964 was associated with biological sex (*P* = 7 × 10^–14^), smoking status measurement (*P* = 1 × 10^–9^), and risk-taking tendency (*P* = 8 × 10^–18^) (Supplementary Table 4). In the “leave-one-out” method, we found that the causal association between beef intake and RA fluctuated slightly after removing three SNPs (rs9379833, rs61853274, and rs7873152) stepwise (Supplementary Figure 1).

## Discussion

In this current study, the stepwise analysis of a cross-sectional study from NHANES 1999–2016 and a two-sample MR study were combined to explore the association between beef intake and the risk of RA. We found a reversed “U” relevance between beef consumption and RA based on NHANES, and a positive association between beef intake and risk of RA by MR. Therefore, the findings indicated that beef intake is suggestively associated with an increased risk of RA.

Previously, a large number of investigators have explored the relationship between different types of meat and the risk of RA. For example, Nguyen et al. conducted a large-scale cohort study including 62,639 participants and suggested that moderate fish consumption was negatively associated with RA risk (41). In addition, a cohort study performed by Sundström et al. in Sweden showed that there was no statistically significant association between poultry intake and risk of RA (42). However, beef is a staple of the American diet, but there was no specific observational study to explore the association between beef intake and RA to date. In our cross-sectional study based on a serial NHANES survey (1999–2016), we found individuals who consumed 50.26–76.50 g of beef per day had a higher risk of RA than those who consumed less than 33.50 g of beef per day. However, except for the third quintile, the risk of RA kept uncertain in other quintiles due to the poor statistical power. Moreover, a reversed “U” relevance between beef consumption and RA was found in dose–response relationship analysis. The non-significant increased risk of more beef consumption might derive from a relatively small sample size but not real effect. Large-sample and well-designed studies should be developed in the future to demonstrate this turning point. Furthermore, the observational studies are easily biased by potential confounding factors and reverse causation (43–45), though we have adjusted age, sex, education level, diabetes, etc. in our analysis. Hence, to further determine the causal association between beef intake and the risk of RA, we conducted a two-sample MR study.

In the MR analysis, we interestingly found that beef consumption is positively associated with RA risk. For MR analysis, it should satisfy three assumptions, which are the premises of causal inference (46). First, there is a robust and strong correlation between IVs and exposure. To ensure this, the loci strongly associated with beef intake reaching the genome-wide significant threshold (*P* = 5×10^−8^) were selected as IVs from a genome-wide association study of 335,576 participants. Second, the IVs must be independent of confounding factors affecting the exposure-outcome relationship. Because genetic alleles are randomly assigned at conception, they could rule out the possibility of the association with confounding factors such as socioeconomic and behavioral factors (47). Third, IVs do not influence the outcome through pathways other than exposure. In the MR-Egger and MR-PRESSO analysis, we found no evidence of directional pleiotropy. For “leave-one-out” analysis, we found the association between beef intake and RA risk was enhanced after excluding rs9972653 and rs4676964, which have the most potential pleiotropic effects. However, the results fluctuated after the exclusion of rs7873152 (*P* = 0.117), rs61853274 (*P* = 0.060), or rs9379833 (*P* = 0.060). These three SNPs were not associated with other traits except beef intake among the European population by searching GWAS Catalog.

A potentially positive association between beef intake and the risk of RA is biologically plausible (14, 48). One explanation is that beef is rich in iron (49), which has been found to be abundant in the rheumatoid synovium mainly in the form of ferritin, contributing to the inflammatory reaction damage (50, 51), such as the promotion of inflammatory mediators including IL-6, IL-8, and IL-1β (52). Another possible explanation is that high collagen in beef increased collagen sensitivity and produced anti-collagen antibodies (21). Besides, the saturated fatty in beef could translocate endotoxin such as lipopolysaccharide toxins and release them into the bloodstream, thus stimulating the immune system and enhancing inflammation (53). High ingestion of fat also promotes the production of endogenous antioxidants, uric acid, and mercaptan, which obviously affects dietary-induced inflammation (54).

There were some limitations that should be noted. First, the imprecise measurement of beef intake along with recall bias and the retrospective diagnosis of RA based on questionnaires might affect the estimation of the association between beef intake and risk of RA in the cross-sectional study. Thus, we conducted an MR study to further clarify the causal relationship. Second, the poor power limited the exploration of a possible non-linear relationships between beef and RA. Third, in view of the data from NHANES and two-sample MR analysis that came from the participants of non-Hispanic white and European descent, it is unknown whether the same results can be applied to other ethnic groups. Forth, the “leave-one-out method” of MR analysis showed an unstable association between beef and RA, which needs to be careful to interpreted this connection. In addition, the limitation of public summary data of other subtypes of red meat, prevented multivariate MR analysis to assess the independent influence of beef intake on RA. Therefore, the role of beef in the development of RA needs further prospective and mechanistic studies to verify.

## Conclusion

Our study suggested a possible causal association between beef intake and risk of RA, while further epidemiologic studies are needed to clarify this suggestive association and the possible dose-response relationship.

## Data availability statement

The original contributions presented in this study are included in the article/Supplementary material, further inquiries can be directed to the corresponding authors.

## Ethics statement

Ethical review and approval was not required for the study on human participants in accordance with the local legislation and institutional requirements. The patients/participants provided their written informed consent to participate in this study.

## Author contributions

ZH and DY conceived and designed the study. WC and KL conducted data analysis and interpreted the results. WC drafted the manuscript. LH, YM, and CW revised the manuscript. All authors read and approved the final manuscript.

## Footnotes

1
https://wwwn.cdc.gov/nchs/nhanes/tutorials

2
https://www.ebi.ac.uk/gwas/

## Abbreviations

CI: confidence interval
FFQ: food frequency questionnaires
FNDDS: Food and Nutrient Database for Dietary Studies
GWAS: genome-wide association study
IVs: instrumental variables
IVW: inverse-variance weighted
MAF: minor allele frequency
MR: Mendelian randomization
MR-PRESSO: Mendelian randomization pleiotropy residual sum and outlier
NHANES: National Health and Nutrition Examination Survey
NHS: Nurses’ Health Study
OR: odds ratio
RA: rheumatoid arthritis
SNPs: single nucleotide polymorphisms.
