# Supplementary material for: Beef intake and risk of rheumatoid arthritis: Insights from a cross-sectional study and two-sample Mendelian randomization
Source: Front Nutr. 2022 Sep 6;9:923472. doi: 10.3389/fnut.2022.923472 (PMC9486088; doi:10.3389/fnut.2022.923472)
Supplement: Supplementary file 1 [file Data_Sheet_1.docx]

Supplementary Material

**Supplementary Table 1.** Detailed information and datasets of exposure or outcome used in the present study

**Supplementary Table 2.** Associations of the instrumental SNPs with beef intake and RA

**Supplementary Table 3.** Ever beef intake and risk of RA in non-Hispanic white based on NHANES 1999-2016

**Supplementary Table 4.** Potential pleiotropic effects of the SNPs used as IVs for beef in European population

**Supplementary Figure 1.** Leave-one-out sensitivity analysis for the association of beef intake with the risk of RA by MR analysis

MR, Mendelian randomization; RA, rheumatoid arthritis.

Supplementary Table 1. Detailed information and datasets of exposure or outcome used in the present study

| Exposure or outcome | PMID | Participants | Source of Population | Web source |
| --- | --- | --- | --- | --- |
| Beef | 32066663 | 335,576 individuals | UK Biobank study | https://www.nature.com/articles/s41398-020-0688-y |
| RA | 24390342 | 14,361 cases and 43,923 controls | European | https://www.nature.com/articles/nature12873 |

Abbreviations: RA, rheumatoid arthritis. UK, United Kingdom.

Supplementary Table 2. Associations of the instrumental SNPs with beef intake and RA

| SNP | Chr | MAF | Beef | | | | | | | | |  | RA | | | |
| --- | --- | --- | --- | --- | --- | --- | --- | --- | --- | --- | --- | --- | --- | --- | --- | --- |
|  |  |  | EA | Beta | | Se | | | *P* | R^2^ | F-statistics |  | EA | Beta | Se | *P* |
| rs4676964 | 3 | 0.227 | C | | -0.015 | | 0.002 | 1.50E-09 | | 8.96E-05 | 30.05 |  | T | 0.010 | 0.020 | 0.690 |
| rs61853274 | 10 | 0.217 | G | | 0.018 | | 0.003 | 2.10E-08 | | 9.38E-05 | 31.49 |  | A | -0.041 | 0.032 | 0.220 |
| rs7873152 | 9 | 0.397 | C | | 0.015 | | 0.002 | 3.50E-10 | | 1.09E-04 | 36.63 |  | T | -0.041 | 0.021 | 0.055 |
| rs9379833 | 6 | 0.358 | C | | 0.020 | | 0.003 | 6.50E-13 | | 1.11E-04 | 37.19 |  | A | -0.020 | 0.018 | 0.370 |
| rs9901521 | 17 | 0.087 | T | | 0.028 | | 0.005 | 4.20E-08 | | 1.18E-04 | 39.50 |  | T | 0.049 | 0.051 | 0.350 |
| rs9972653 | 16 | 0.317 | G | | 0.014 | | 0.003 | 7.40E-09 | | 1.54E-04 | 51.82 |  | T | 0.000 | 0.015 | 0.850 |

| Supplementary Table 3. Ever beef intake and risk of RA in non-Hispanic white based on NHANES 1999-2016 |
| --- |

| Beef intake | Case | Control |  | Model 1 | |  | Model 2 | |
| --- | --- | --- | --- | --- | --- | --- | --- | --- |
|  |  |  |  | OR | 95% CI | | OR | 95% CI |
| No | 647 | 6200 |  | Ref | | | Ref | |
| Yes | 259 | 2512 |  | 1.09 | 0.88 to 1.35 | | 1.05 | 0.85 to 1.29 |

| Model 1: adjust for age, sex, education, poverty-income ratio, marriage.  Model 2: adjust for age, sex, education, poverty–income ratio, marriage, smoking, alcohol drinking, history of diabetes, BMI. |
| --- |

| **Supplementary Table 4.** Potential pleiotropic effects of the SNPs used as IVs for beef in European population |
| --- |

| SNP | Trait (s) | P-value | PubMed ID | |  | Location |
| --- | --- | --- | --- | --- | --- | --- |
| rs4676964 | Risk-taking tendency (4-domain principal component model) | 8 × 10^-18^ | 30643258 |  | | 3:70985597 |
|  | Biological sex | 7 × 10^-14^ | 33888908 |  | | 3:70985597 |
|  | Smoking status | 1 × 10^-9^ | 30595370 |  | | 3:70985597 |
| rs61853274 | NA | NA | NA |  | | NA |
| rs7873152 | NA | NA | NA |  | | NA |
| rs9379833 | NA | NA | NA |  | | NA |
| rs9901521 | Neuroticism | 4 × 10^-10^ | 29942085 |  | | 17:81124352 |
| rs9972653 | Body fat mass | 2 × 10^-14^ | 28552196 |  | | 16:53780451 |
|  | Lean body mass | 5 × 10^-9^ | 28552196 |  | | 16:53780451 |
|  | Heel bone mineral density | 7 × 10^-10^ | 28869591 |  | | 16:53780451 |
|  | Relative sugar intake | 1 × 10^-11^ | 32393786 |  | | 16:53780451 |
|  | Walking pace | 9 × 10^-16^ | 33128006 |  | | 16:53780451 |
|  | Heel bone mineral density | 8 × 10^-12^ | 30048462 |  | | 16:53780451 |
|  | Serum albumin levels | 2 × 10^-9^ | 34594039 |  | | 16:53780451 |
|  | Neutrophil count | 2 × 10^-10^ | 34594039 |  | | 16:53780451 |
|  | White blood cell count | 4 × 10^-15^ | 30595370 |  | | 16:53780451 |
|  | Pure non-grapefruit juice consumption | 1 × 10^-9^ | 31046077 |  | | 16:53780451 |


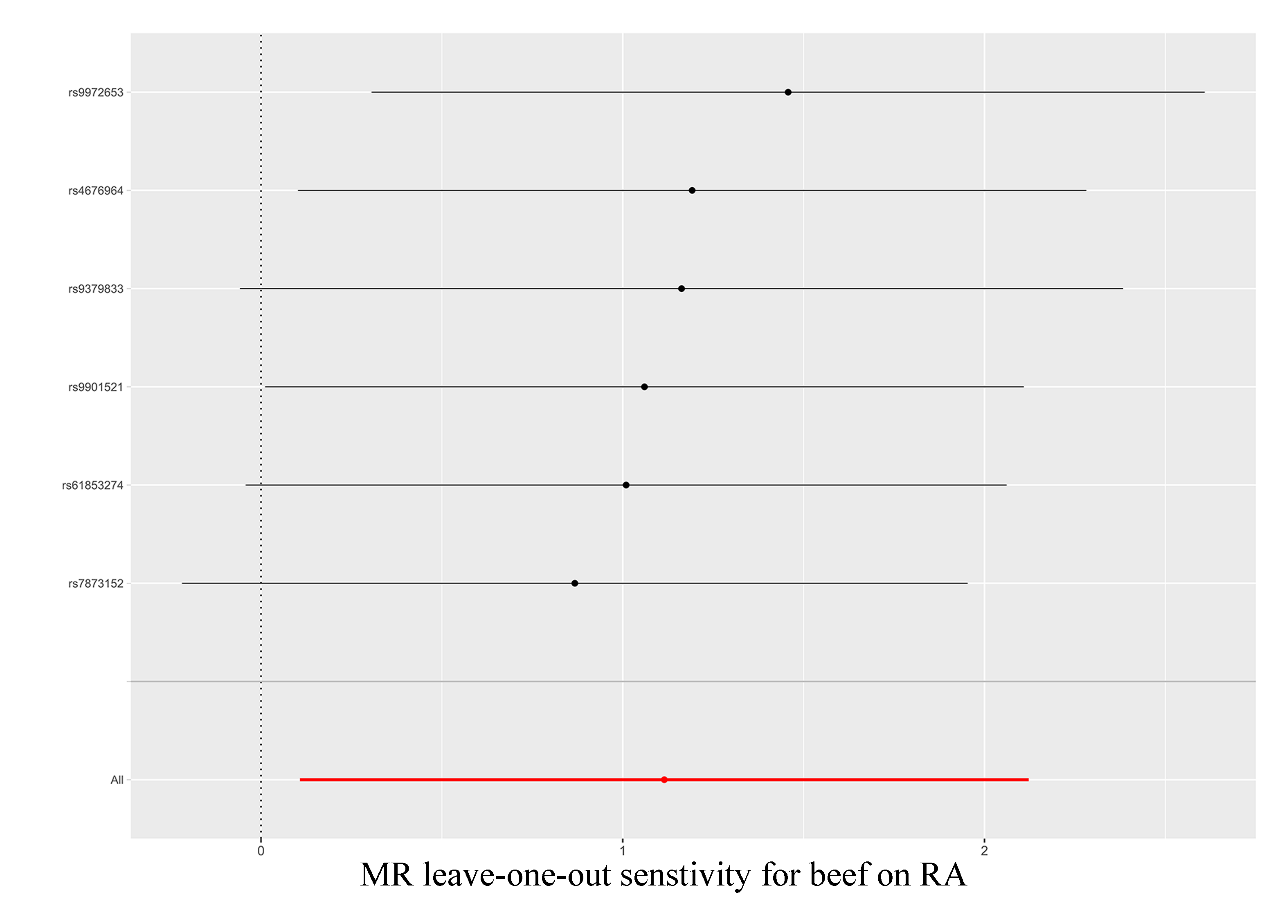
**Supplementary Figure 1.** Leave-one-out sensitivity analysis for the association of beef intake with the risk of RA by MR analysis
MR, Mendelian randomization; RA, rheumatoid arthritis.
